# An Institute of General Pathology and Preventive Medicine

**Published:** 1920-03-13

**Authors:** 


### MEMORIAL TO SIR WILLIAM OSLER.

### An Institute of General Pathology and Preventive Medicine.

There is no place in England that could so well enshrine the memory of Sir William Osler than Oxford, that "glorious city, whose charm none can tell." Whether his own College of Christ Church, that magnificent foundation planned by Cardinal Wolsey, and established by Henry VIII., or the Bodleian, beloved as a dear child by so ardent a bibliophile, in the Museum, at which all the scientific studies of the university are taught and investigated, or at the Radcliffe Infirmary, where week by week the world-famed physician led his class, should be chosen to bear his name for ever, was the object of a meeting at the Museum on Saturday, March 6 last. The meeting was called by the members of the Faculty of Medicine, and was presided over by the Vice-Chancellor of the University (Dr. Blakiston). Among the company present were several Heads of Houses, the Regius Professor of Physic at Cambridge (Sir Clifford Allbutt), the new Regius at Oxford (Sir Archibald Garrod), Sir William Church (ex-President of the Royal College of Physicians), Sir Norman Moore, Sir George Newman, Surgeon-General Sir Frederick Bradshaw, and many other distinguished members of the University and the medical profession.

It had been hoped that the American Ambassador, the Ambassador to the United States, and the High Commissioner for Canada would have been able to be present, but their engagements unfortunately kept them away, although they expressed themselves in sympathy with the proposals to be made.

### Respect and Affection.

The Vice-Chancellor in a brief opening speech said that they had met out of the respect and affection they felt for their late Regius Professor; and not only was this felt by them but by other universities, by the London hospitals, and by other continents. Whether his name would be associated with the future history of medicine in Oxford, would depend upon the result of the meeting held that day.

Sir Clifford Allbutt then submitted a resolution that "the distinguished services of the late Sir William Osler were deserving of a permanent memorial in Oxford." He said that it was a great privilege to speak first on this occasion and to speak as he did representing others in his own university, who were concerned with this cause as deeply as anyone in Oxford. It was not the occasion for any eulogy; the learning of Sir William, his charm of character were so well known as to need no words from him. Sir William possessed a wonderful experience which brought to him the affection and intimacy of the friendships of men throughout the whole world and which caused every man to find in him a feeling and sympathetic friend. In art, in science, in humanity, he was an "aerial," who in a wide circle brought a sense of unity over the world and became a bond for mankind in divers countries. None who had once known him could ever forget him. Oxford could not forget him, and he could never be forgotten—but that the future generations should have some token of the affection of these days it was proposed in view of the intimate association of Sir William Osler's life-work with the study of the origin and prevention of disease, that the most appropriate form of memorial would be an "Osler Institute of General Pathology and Preventive Medicine."

Sir Herbert Warren, President of Magdalen College, seconded the resolution, and spoke of that first and important trait in Sir William Osler's character—his optimistic belief in human nature. He knew so much and did so much, and if anything served to describe Sir William's work more than anything else it would be found in the oath which every physician had of old to take and to be found in Hippocrates.

### The Feeling at Oxford.

Oxford suited him as he suited Oxford, and whatever section of her life he took up he builded it more broadly and very wisely. Any memorial which, therefore, was to be formed should be made as worthy as we possibly could make it.

Sir William Church and Professor Arthur Thomson then supported the proposal for an Osler Institute, and the Dean of Christ Church (Dr. Strong) then submitted a resolution that "a general and executive committee be appointed to issue an appeal and collect funds."

All the proposals were passed unanimously, and Professor Gunn, who is acting as honorary secretary, said that a committee had been appointed in America to co-operate with the Oxford Committee, and that it was hoped a similar committee could be set going in Canada for the purpose of making this scheme practicable and worthy of the great name it bears.

This proposed memorial will link up the work of the University with that of the Radcliffe Infirmary, and do something for a side of medicine which for so many years has been neglected in the hospital practice of the country. It is hoped that it will meet with most generous support.

